# Immunomodulatory effect from ethanol extract and ethyl acetate fraction of *Curcuma heyneana* Valeton and Zijp: Transient receptor vanilloid protein approach

**DOI:** 10.1016/j.heliyon.2023.e15582

**Published:** 2023-04-20

**Authors:** Fifteen Aprila Fajrin, Melanny Ika Sulistyowaty, Mohammad Labib Ghiffary, Swara Adla Zuhra, Wulan Rosa Panggalih, Dwi Koko Pratoko, Fransiska Maria Christianty, Katsuyoshi Matsunami, Anastasia Wheni Indrianingsih

**Affiliations:** a Clinical and Community Department, Faculty of Pharmacy, Universitas Jember, 68121, Indonesia; b Preclinical Pharmacology Research Group, Faculty of Pharmacy, Universitas Jember, 68121, Indonesia; c Department of Pharmaceutical Sciences, Faculty of Pharmacy, Universitas Airlangga, 60115, Indonesia; d Pharmaceutical Chemistry Department, Faculty of Pharmacy, Universitas Jember, 68121, Indonesia; e Graduate School of Biomedical and Health Sciences, Hiroshima University, 1-2-3 Kasumi, Minami-ku, Hiroshima, 734-8553, Japan; f Research Center for Food Technology and Processing, National Research and Innovation Agency (PRTPP BRIN), Yogyakarta 55861, Indonesia

**Keywords:** Immunomodulator, Carbon clearance, *Curcuma heyneana*, Phagocytic index, Leukocyte cell count, Mice

## Abstract

This study aims to discover the immunomodulatory potential of the ethanol extract (EE) and the ethyl acetate fraction (EAF) of *Curcuma heyneana* Valeton and Zijp (Indonesian name: temu giring) rhizome using mice models. The affinity of the curcuminoid (curcumin, dimethoxy-, and bisdemethoxy-) through the Transient Receptor Potential Vanilloid 1 (TRPV1) was determined using Mollegro molecular docking in silico. The curcuminoid concentration of the EE and EAF of *C. heyneana* rhizome were determined using thin-layer chromatography densitometry. *In vivo* studies in mice models were conducted using the carbon clearance method to determine the phagocytosis index, and the number of leukocytes in the blood and spleen. Forty mice were divided into eight groups, including negative control (given 1% CMC-Na), positive control (given Stimuno Forte® suspension at a dose of 6.5 mg/kg BW), three groups given the EAF of *C. heyneana* rhizome extract at a dose of 125 mg/kg BW, 250 mg/kg BW, and 500 mg/kg BW, respectively, and three groups were given EE of temu giring rhizome extract with doses of 125 mg/kg BW, 250 mg/kg BW, and 500 mg/kg BW, respectively. E.E. and E.A.F. of *C. heyneana* (temu giring) rhizome extract contained dimethoxy curcumin (0.176 ± 0.01 and 4.53 ± 0.02 %b/b) greater than another curcuminoid, bisdemetoxy curcumin and curcumin. EE at 125 mg/kg BW and EAF dose at 500 mg/kg B W. of temu giring rhizome have immunostimulant activity with a phagocytosis index value of >1 compared to the negative control (p < 0.05). Additionally, both increase the number of lymphocytes, monocytes, and neutrophil cells in peripheral blood and spleen compared to the negative control (p < 0.05). Their activity was seen as similar to the positive control. Therefore, the EE of *C. heyneana* rhizome has immunostimulant activity, and the EAF of *C. heyneana* rhizome has immunosuppressant activity at 125 mg/kg BW and immunostimulant at a higher dose. The activity of temu giring as an immunomodulator was associataed with its affinity to TRPV1.

## 1 Introduction

The immune system is a complex mechanism that occurs as the body responds to foreign substances or infections. The immune system is categorized into the innate immune system (non-specific immune system) and the adaptive immune system (specific immune system) [1]. Some disorders involve the uncontrolled immune system, such as autoimmune disease, immunodeficiency disorders, and hypersensitivity [2]. The disease can be healed by immunomodulators which are defined as drugs or substances that work by modulating the function and activity of the immune system. Immunomodulators are divided into three groups: immunoadjuvants, immunostimulants, and immunosuppressants [3].

Corticosteroids (glucocorticoids), cyclosporine, and azathioprine are examples of immunosuppressant drugs that work by inhibiting the immune system [4]. Other drugs, such as levasimol and isoprinosine, are examples of immunostimulant drugs that work by activating the immune system [5]. However, long-term use of these drugs will cause unwanted side effects, such as gastric ulcers, osteoporosis, psychological changes, muscle atrophy, and increased intraocular pressure [6]. Hence, drugs derived from plants were developed.

A previous review [7] found many journals discussing plants from the Curcuma genus that have immunosuppressants and immunostimulants activities, such as *Curcuma longa* Linn, *Curcuma zanthorrhiza* Roxb, *Curcuma aeruginosa* Roxb, *Curcuma zedoaria* (Christm.) Roscoe, and *Curcuma mangga* Valeton and Zijp. Many components in these plants are responsible for their activity as immunosuppressants and immunostimulants. One of the essential components of the Curcuma genus that plays a role in immunomodulators is curcumin. Curcumin has been widely reported as having strong antitumor, antioxidant, anti-inflammatory, and immunomodulating activities [8].

Another plant from the Curcuma genus, such as *Curcuma heyneana* Valeton and Zijp, also known as temu giring (Indonesian name), was previously reported to contain curcuminoids and sesquiterpenes [9]. *C. heyneana* (temu giring) is a typical native plant of Indonesia that contains many chemical compounds, such as essential oils, saponins, flavonoids, tannins, starch, fat, resin, and yellow dye [10,11]. A previous study [12] revealed flavonoids, curcumin, triterpenoids, vitamin C, vitamin E, and catechins as compounds that play a role in immunomodulators in natural ingredients. The *C. heyneana* rhizome plant may have the potential as an immunomodulator based on the content of these compounds.

Our previous studies on *Zingiber officinale* Roxb extract revealed its activity as an analgesic and anti-inflammatory in painful diabetic neuropathy through the Transient Receptor Potential Vanilloid 1 (TRPV 1) [13]. Generally, TRPV1 was previously known as one of the receptors which are responsible for cation influx in many cells [14]. TRPV1 is found in large concentrations in the nervous system, such as the spinal cord and dorsal root ganglia. Additionally, TRPV1 is expressed in the kidney, pancreas, testes, uterus, spleen, stomach, small intestine, lung, and liver mucous gland [15]. Lately, TRPV1 was reported to play a role in the immune system [16]. Some studies revealed how TRPV1 is responsible for the immunomodulatory effect although it remained debated. Thus, exploring a new treatment as an immunomodulator with a TRPV1 activity approach is interesting. Therefore, our study aimed to determine the affinity of curcuminoid (curcumin, dimethoxy-, and bisdemethoxy-) using in silico study with Mollegro molecular docking and revealed the immunomodulatory activity using a mice model.

## 2 Materials and methods

### 2.1 In-silico study of curcuminoid to TRPV1

#### 2.1.1 Hardware and software

The hardware used is ACER Aspire 3 A314-41 notebook with AMD A9 9420 @ 3.6 GHz processor, and Windows 10 O.S., 64 bit, 4 GB RAM. The Molegro Virtual Docker 6.0 (free trial) and ChemOffice 2010, consisting of ChemBio Draw Ultra 12.0 and ChemBio3D Ultra 12.0, were used in this study.

#### 2.1.2 Molecular structure and optimization

The molecular structure of curcumin and its derivatives were drawn using ChemBio Draw Ultra 12.0 2010. Furthermore, optimization was conducted on the molecular structure geometry using MM2 tools on ChemBio3D ultra 12.0 2010. The TRPV1 receptor complex structure with capsazepine (PDB ID 5IS0) was obtained through Protein Data Bank (http://www.rscb.org).

#### 2.1.3 Docking method validation

**Fig. 1:**
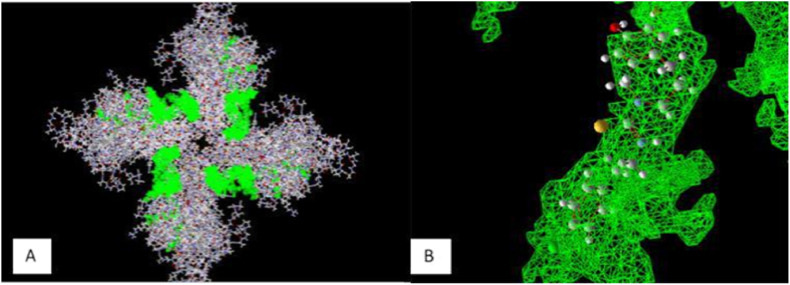
Interaction of TRPV1 and Capsazepine in Cavity 5. A. 5IS0 after detecting cavities; B. Native ligand.

Docking process validation performed by a re-docking method using Molegro Virtual Docker 6.0. Validation was conducted on the active site of the result of 5IS0 crystallography. The 6 ET 801 (D) ligand in the conformation found in the complex structure of the crystal ligand (Fig. 1B) was set into the active side of the cavity (cavity 5 vol 225.792).

#### 2.1.4 Molecular docking

The molecular docking process was conducted to determine the binding location of the ligand to the receptor. The test ligands were included curcumin, dimethoxy curcumin, and bisdemethoxycurcumin. The parameters measured in the docking process are the involved energy value (the MolDock score and the rerank score). A parameter that often used is the rerank score to calculate the strength of ligand-receptor bonds. Three replications of each compound did the process of re-docking and docking. Additionally, the hydrogen bond determination was evaluated.

#### 2.1.5 Prediction of toxicity

All the chemical structures of curcumin and its derivates were prepared in the simple molecular-input entry row system. Each was predicted online to find their toxicity at http://biosig.unimelb.edu.au/pkscm/prediction and compared using the lethal dose 50 (LD50) and Lowest Observed Adverse Effect Level (LOAEL) values.

### 2.2 Preparation of the extract and fraction of temu giring

*C. heyneana* (temu giring) rhizome was obtained from the Purwodadi Botanical Gardens Center in March 2021. Afterwards, we extract the rhizome of *C. heyneana* as much as 3 kg with ethanol 96% by maceration for three days and obtained as much as 265 g of ethanol extract of *C. heyneana* (EETG). Then, the extract was mixed with water and fractionated gradually using ethyl acetate and named as ethyl acetate fraction of *C. heyneana* (EAFTG).

### 2.3 Curcuminoid analysis using thin layer chromatography densitometry

A 1000 ppm standard stock solution was prepared by weighing 1 mg standard curcumin and dissolved by ethanol up to 1 mL. Series concentrations were prepared to obtain 50, 100, 150, 200, and 250 ppm. The sample solution was prepared by weighing the ethyl acetate fraction and ethanol extract of 500 mg dissolved in 5 mL of ethanol p.a. Later; the solution was sonicated for 10 min.

The standard curcumin and sample solution were spotted on the T.L.C. plate. The mobile phase used was a mixture of chloroform: methanol (9.6:0.4) (v/v). The T.L.C. plate was ejected and eluated into the chamber, saturated with the mobile phase. The measurement of curcumin levels was determined by densitometry at 424 nm.

### 2.4 Preparation of test animal

Mice were first adapted for one week in cages with feeding and ventilation, which were always kept clean to keep them healthy. Healthy mice were characterized by increased body weight and active movement. The animal testing procedure was conducted following the Ethical Committee of Medical Research, University of Jember, Indonesia, with the supervision of Prof. drg. I Dewa Ayu Ratna Dewanti, M.Si, with document number 1218/UN25.8/KEPK/DL/2021.

### 2.5 Preparation of the suspension test

This study used a dose of Stimuno Forte® at 6.5 mg/kg BW [17]. The EETG and EAFTG suspension was made in several doses (125 mg/kg BW, 250 mg/kg BW, and 500 mg/kg BW) of 1% of CMC-Na. Carbon suspension as an inductor was made in 16% concentration in 0.9% physiological NaCl solution [18].

### 2.6 Study of immunomodulatory effect using mice model

This study used 40 Balb/C strain mice divided into eight groups. Each group consisted of five mice and was given different treatments.

a. The negative control group (NCG) was given CMC-Na 1%.
b. The positive control group (PCG) was given Stimuno at a 6.5 mg/kg BW.
c. Test group 1 (EETG 125) was given EETG at 125 mg/kg BW.
d. Test group 2 (EETG 250) was given EETG at 250 mg/kg BW.
e. Test group 3 (EETG 500) was given EETG at 500 mg/kg BW.
f. Test group 4 (EAFTG 125) was given EAFTG at 125 mg/kg BW.
g. Test group 5 (EAFTG 250) was given EAFTG at 250 mg/kg BW.
h. Test group 6 (EAFTG) was given EAFTG at 500 mg/kg BW.

Each group was given orally once a day for six consecutive days. On the seventh day, the mouse's tail was cut, and the blood was collected on a plate with Na EDTA added. Then, 25 L of blood was taken and lysed in 4 mL of 1% acetic acid. The first blood (before carbonation) was used as a blank (0^th^ min). Then, the carbon suspension was intravenously injected into the tail as much as 0.1 mL. A 25 μL of mouse blood was taken 5 and 15 min after injection. Each blood was lysed in 4 mL of 1% acetic acid, then vortexed, and the absorbance was measured using a UV–Vis spectrophotometer at a wavelength of 640.5 nm. This method was referred from previous methods with some modifications [19,20].

### 2.7 Calculation of constant and phagocytosis index

The carbon clearance test was determined by calculating the phagocytosis constant (K) and phagocytosis index (IF) using the following equations (1), (2)), respectively [21].

(1) $$\left(K\right)=\frac{lnOD1-\ln\;OD2}{t2-t1}$$

K: Phagocytosis constant
OD1: Absorbance at minute 5
OD2: Absorbance at minute 15
T: Time (5, 15)

(2) $$\left(IF\right)=\frac{Mice\;Constant\;Z}{Phagocytosis\;Constant\;of\;Negative\;Control\;Mice}$$

IF: The phagocytosis index of each test group was compared with the control group.

Z Mice: Mice that have been treated and determined the value of the phagocytosis constant.

### 2.8 Assessment of the number of leukocytes, lymphocytes, monocytes, and neutrophils

Blood from the tail was dripped as much as 1 mL on the object glass and then flattened with a cover glass to obtain a homogeneous blood layer. Spleen samples were previously suspended with 3 mL of phosphate buffer (pH 7.4) and as much as 20 μL were taken, then, dripped with methanol and left for 5 min after drying. All samples were added with Giemsa solution, incubated for 20 min, and then rinsed with running distilled water and air-dried. The samples were examined under a light microscope at 1000× magnification using immersion oil [19]. Each cell of lymphocytes, monocytes, and neutrophils was counted to find 100 leukocytes [22]. Then the percentage of the number of lymphocytes, monocytes, and neutrophils from the 100 leukocytes was calculated using equations (3), (4), (5), respectively [23].

(3) $$Lymphocyte=\frac{Lymphocyte\;count}{100\;leukocytes}\times 100\%$$

(4) $$Monocytes=\frac{Monocytes\;count}{100\;leukocytes}\times 100\%$$

(5) $$Neutrophil=\frac{Neutrophil\;count}{100\;leukocytes}\times 100\%$$

### 2.9 Data analysis

All data were provided into mean ± S.D. The rerank score, phagocytosis index, and the number of leukocytes, neutrophils, and lymphocytes were analyzed using one-way ANOVA with a 95% interval. To determine the differences between groups, we used L.S.D. (Least Significance Difference). The p-value <0.05 was concluded as significantly different.

## 3 Results and discussion

### 3.1 Docking method validation

The docking method validation results are shown in Fig. 1. Three replications of the re-docking revealed root mean square deviation (RMSD) values of 1.564 Å, 1.734 Å, and 1.806 Å with an average of 1.701 Å. The docking method was valid if the value of RMSD was less than 2.0 Å [24]. The 5IS0 receptor could be used for docking because the atoms in the ligand (of the re-docking result) did not differ too far from the crystallographic position ligand as seen in Fig. 1A [25]. The native ligand was observed in cavity 5 as seen in Fig. 1B. The other parameters observed in the validation process are the MolDock score, the rerank score, and the native ligand.

### 3.2 Model interaction between curcumin and its derivatives with TRPV1 receptors

The docking analysis of the test ligands with their receptors (Table 1) was calculated from stable conformation as a rerank score value described as the ligand's affinity binds with the receptor. Rerank score is a value that reflects the bond energy needed to form a bond between a ligand and its receptors, thus the predictable activity of a compound. The smaller the rerank score value, the greater the ligand bond with the receptor.

**Table 1:** Rerank score of the curcuminoid into TRPV1.

Compound	Log P*	MolDock Score	Rerank Score	Average of Rerank Score
Curcumin	2.17	−129.225 −134.135 −130.205	−112.341 −116.009 −112.543	−113.631 ± 2.061^a^
Dimethoxy curcumin	2.29	−128.314 −120.576 −129.243	−108.746 −101.072 −109.973	−106.597 ± 4.824^a^
Bisdemethoxy curcumin	2.42	−120.822 −121.070 −116.180	−102.183 −102.092 −98.933	−101.069 ± 1.851^b^
Capsazepine (native ligand)	4.21	−112.409 −110.442 −113.662	−98.352 −96.483 −98.487	−97.774 ± 1.120^b^

*log P values were prepared using ChemBio Draw Ultra 12.0. The different superscript letters showed significant differences between groups using One Way ANOVA, 95% and L.S.D. posthoc.

**Fig. 2:**
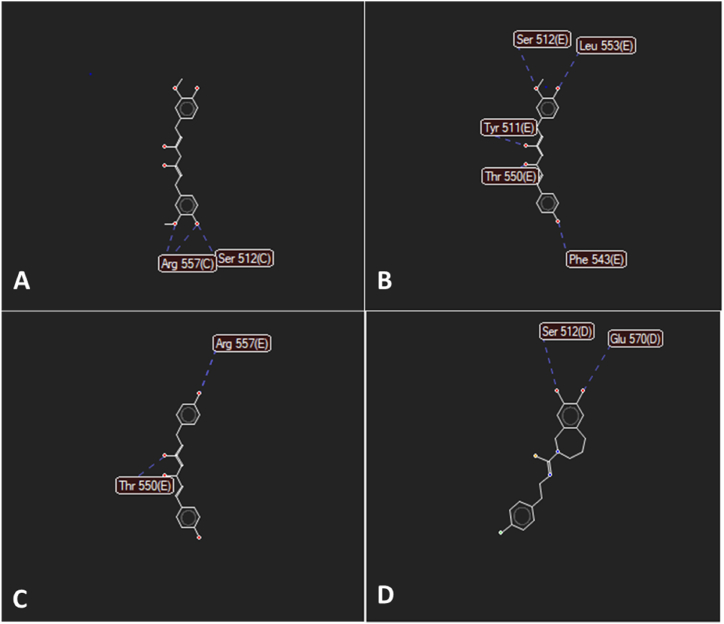
The hydrogen bonds of the test ligands and capsazepine with TRPV1. A. curcumin; B. dimethoxycurcumin; C. bisdemethoxycurcumin; D. capsazepine.

Table 1 shows the averages of the three replications: curcumin (−113.631 ± 2.062), dimethoxy curcumin (−106.597 ± 4.824), bisdemethoxycurcumin (−101.069 ± 1.851); and capsazepine (−97.774 ± 1.120). The rerank scores of curcumin and its derivatives were lower than capsazepine, which is a TRPV1 receptor antagonist, indicating that curcumin and its derivates had better binding affinity than the native ligand. However, curcumin was remained a compound with the best affinity and was not significantly different from dimethoxy curcumin toward TRPV1. The prediction results of hydrogen bonds of capsazepine, curcumin, and its derivatives are shown in Table 2 and Fig. 2.

**Table 2:** Hydrogen bonds of the curcuminoid into TRPV1.

	Curcumin	Dimethoxy curcumin	Bisdemethoxycurcumin	Capsazepine
Ser 512	+	+	−	+
Glu 570	−	−	−	+
Leu 553	−	+	−	−
Tyr 511	−	+	−	−
The 550	−	+	+	–
Phe 543	−	+	−	−
Arg 557	+	−	+	−

Curcumin and dimethoxy curcumin had higher activity than capsazepine as a native ligand toward TRPV1 using in silico study. Both had hydroxyl (–O.H.) and methoxy (–OCH_3_) groups. Hydroxyl (–O.H.) and methoxy (–OCH_3_) groups were aromatic group substituents (of curcumin and its derivatives), which played an essential role in biological activity. They played a role in inhibiting the activity against the TRPV1 receptor. Bisdemethoxycurcumin loses two methoxy groups, according to its structure, thereby decreasing the activity.

All curcumin derivatives except bisdemethoxycurcumin had hydrogen bonds on Ser 512 (Fig. 2A–D). Hence, bisdemethoxycurcumin had a lower affinity compared to the other derivative. A compound without a hydrogen bond with Ser 512 will show decreased activity. Curcumin had two hydrogen bonds on Ser 512 and Arg 557 (the –O.H. and –OCH_3_ groups; Fig. 2A). Dimethoxycurcumin (Fig. 2B) had hydrogen bonds at Ser 512 (–OCH_3_ group), Leu 553; Tyr 511; Phe 543 (–O.H. group), and Thr 550 (–C]O). Capsazepine (Fig. 2D) had hydrogen bonds at Ser 512 and Glu 570 (–O.H. group). Bisdemethoxycurcumin had the same bond with curcumin at Arg 557 although it did not bond with Ser 512 (Fig. 2C). Thus, bisdemethoxycurcumin still had better activity than capsazepine. Therefore, the bond in the amino acid Ser 512 was significantly influenced by the presence of antihyperalgesic activity in curcumin derivatives and capsazepine. The effect was more significant than the bond with Arg 557.

### 3.3 Toxicity prediction of curcuminoid with TRPV1

Evaluating and understanding the pharmacokinetics and toxicity properties is more accessible using pkCSM. Our result revealed that curcumin was the safest compared to another derivate with LD50 at 2.193 mol/kg and LOAEL at 1.708 log mg/kg BW/day (Table 3).

**Table 3:** Result of the toxicity prediction using pkCSM.

	AMES toxicity	Max Tolerated dose (log mg/kg/day)	L.D. (mol/kg)	LOAEL (log mg/kgbw/day)	Hepato-toxicity	Skin sensitization
Curcumin	No	0.051	2.193	1.708	No	No
Dimethoxy curcumin	No	0.018	2.157	1.65	No	No
Bisdemethoxy curcumin	Yes	0.093	1.886	1.676	No	No
Capsazepine	No	−0.124	2.368	1.401	No	No

LD_50_ was defined as oral rat acute toxicity. LOAEL was defined as oral rat chronic toxicity-lowest observed adverse effect.

Moreover, we could relate the compounds' partition coefficient or log P-value with the affinity predictions. Compounds have high lipophilicity because the loss of –O.H. bonds could reduce their affinities to the receptor, so their activities also decreased. Based on Lipinski's “rules of five,” log P has a role in controlling the compounds' lipophilicity and thus influencing the pharmacokinetics and ADMET properties of the compounds. Log P < 5 is suitable for the physiological transport of drugs, while log P > 3 can increase the likelihood of toxicity [26]. This result was supported by our toxicity prediction analysis using pkCSM. However, some literature suggested a good log P between 1 and 3. This value was affected by curcumin and dimethoxy curcumin. Therefore, affinity and curcumin activity, dimethoxy curcumin, and bisdemethoxycurcumin were better than capsazepine.

### 3.4 Curcuminoid analysis using T.L.C

Quantitative analysis of the ethyl acetate fraction of temu giring shows the results of the levels as shown in Table 4. Curcuminoid content in the extract showed a higher result than in the fraction.

**Table 4:** Curcuminoid content in extract and fraction of C. heyneana (temu giring).

Compound	Curcuminoid content (% w/w ± SD)		
	Curcumin	Demethoxy curcumin	Bisdemethoxy curcumin
Extract	0.026 ± 0.001	4.533 ± 0.022	0.401 ± 0.003
Fraction	0.026 ± 0.001	0.176 ± 0.001	0.141 ± 0.004

### 3.5 Effect of EETG and EAFTG treatment in phagocytosis index of mice

Carbon cleaning methods were performed to calculate the constant and phagocytosis index. The phagocytosis index will be directly proportional to the phagocytosis constant, which means the greater the value of the phagocytic constant and index, the faster the phagocytic process conducted by phagocytic cells in eliminating carbon in the bloodstream. The comparison of the mean, standard deviation (SD), and statistical analysis of the phagocytic index in each group is shown in Table 5.

**Table 5:** Comparison of the mean, S.D., and statistical analysis of phagocytosis index in each group.

Group	Phagocytosis Index (Mean ± S.D.)
Negative Control (CMC-Na 1%)	1.000 ± 0.000^a^
Positive Control (Stimuno 6.5 mg/kg BW)	4.146 ± 0.936^b^
EETG rhizome dose 125 mg/kg BW	1.998 ± 0.761^c^
EETG rhizome dose 250 mg/kg BW	3.812 ± 1.948^b^
EETG rhizome dose 500 mg/kg BW	4.242 ± 1.190^b^
EAFTG rhizome dose 125 mg/kg BW	0.572 ± 0.277^d^
EAFTG rhizome dose 250 mg/kg BW	2.220 ± 1.208^a^
EAFTG rhizome dose 500 mg/kg BW	4.317 ± 1.738^b^

The different superscript letters showed significant differences between groups using One Way ANOVA, 95% and L.S.D. posthoc.

The value of the phagocytosis index in the test group of the EETG at all doses (125 mg/kg BW, 250 mg/kg BW, and 500 mg/kg B.) was significantly different (p < 0.05) with the NCG and EETG (250 mg/kg BW, and 500 mg/kg BW) was not significantly different (p > 0,05) with the PCG. Meanwhile, the value of the phagocytosis index in the EAFTG at 125 mg/kg BW was lower than the negative control and significantly different (p < 0.05). The EAFTG at 250 mg/kg BW was not significantly different (p > 0.05) from the NCG, and the EAFTG at 500 mg/kg BW was not significantly different (p > 0.05) from the positive control.

Our study used a carbon cleansing assay to evaluate the effects of drugs and phytoconstituents on the reticuloendothelial system (R.E.S.) [27]. The RES, also known as the mononuclear phagocyte system, consists of monocytes, macrophages, and dendritic cells in tissues throughout the body. These cells play a role in the phagocytosis process [28]. The carbon will be eliminated by the RES through phagocytosis after intravenously injecting the colloidal carbon particles into the blood. The rapid removal of carbon particles from the blood has been associated with increased phagocytic activity [27]. The value of the phagocytosis constant indicates the speed of the phagocytosis process. The value of the phagocytosis index shows the magnitude of the phagocytic activity of phagocytic cells against carbon particles which are considered antigens [29].

Therefore, the higher the dose of EETG and EAFTG, the higher the phagocytosis index value. According to a previous study [[26], [28], [29]], the phagocytic index value (IF of <1) has immunosuppressant activity, and the phagocytic index (IF of >1) has immunostimulating activity. The phagocytic index value produced (IF of >1) could provide immunostimulant activity at all EETG doses. Meanwhile, the phagocytosis index produced by the EAFTG at 125 mg/kg BW (IF < 1) gave immunosuppressant activity, and increased dose of EAFTG would give immunostimulant activity (IF of >1). Therefore, EETG doses at 125, 250, and 500 mg/kg BW have immunostimulant activity, while EAFTG has immunosuppressant activity (dose of 125 mg/kg BW) and immunostimulant (doses of 250 and 500 mg/kg BW).

Increasing the value of the phagocytic index in each test group can provide an overview of the effect EETG and EAFTG on the phagocytic activity of the immune system. This is due to the presence of curcumin, the main constituent of the *C. heyneana* rhizome, that acts as an immunostimulant [9,30]. Curcumin is known to influence innate and adaptive immunity by modulating the function of immune cells, including neutrophils, macrophages, monocytes, natural killer cells (N.K. cells), dendritic cells (D.C.), T cells, and B cells [31,32]. Research conducted by Safia and Samia (2016) [33] on the effect of temulawak extract on macrophage phagocytic activity using the carbon clearance method revealed that the increase in the phagocytic index value at each dose was caused by the presence of curcumin which can increase phagocytic activity of macrophages by stimulating the RES. Curcumin has inconsistent immunomodulatory activity because it can stimulate the immune system or reduce the work of the immune system [34]. *C. heyneana* rhizome, in addition to curcumin, contains flavonoids that will work when the activity of the immune system decreases. Then flavonoids will send intracellular signals to cell receptors to increase their activity. However, if the opposite occurs, then flavonoids will provide benefits for reducing the work of the immune system so that it functions as a balancer for the immune system [35].

### 3.6 The effect of EETG and EAFTG treatment on leukocytes in the peripheral blood and spleen

**Fig. 3:**
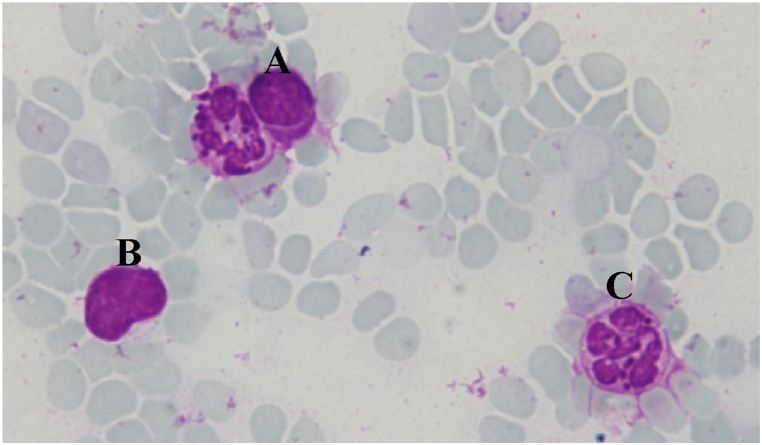
Overview of lymphocytes (A), monocytes (B), and neutrophils (C) using a light microscope with 100× magnification and Giemsa stain.

Leukocytes such as lymphocytes, monocytes, and neutrophils were counted under a microscope with 100× magnification using immersion oil as an explanatory and Giemsa as a dye (Fig. 3). The effect of EETG and EAFTG administration on leukocytes were shown in Table 6.

**Table 6:** Comparison of the number of lymphocytes, monocytes, and neutrophils in peripheral blood and spleen in mice after EETG and EAFTG treatments.

Group	Lymphocyte (% ± SD)		Monocyte (% ± SD)		Neutrophil (% ± SD)	
	Blood	Spleen	Blood	Spleen	Blood	Spleen
Negative Control (CMCNa 1%)	52.8 ± 4.0^a^	99.6 ± 2.9^a^	5.8 ± 1.3^a^	4.6 ± 1.3^a^	21.6 ± 3.6^a^	16.2 ± 3.8^a^
Positive Control (Stimuno 6.5 mg/kgBW)	60.6 ± 7.5^a.b.c^	145.6 ± 7.4^d^	13.2 ± 2.4^c^	6.4 ± 1.1^a^	31.0 ± 5.5^b.c^	21.2 ± 1.3^a.b.c^
EETG 125 mg/kg BW	56.2 ± 6.9^a.b^	113.8 ± 4.2^b^	11.8 ± 2.4^b.c^	4.8 ± 1.9^a^	29.6 ± 3.8^b.c^	16.2 ± 3.3^a^
EETG 250 mg/kg BW	64.0 ± 4.5^b.c^	152.6 ± 9.4^d^	13.2 ± 2.9^c^	7.0 ± 1.2^a.b^	32.2 ± 5.0^c^	22.8 ± 2.8^b.c.d^
EETG 500 mg/kg BW	68.4 ± 4.3^c^	167.8 ± 7.2^e^	20.8 ± 3.2^d^	10.2 ± 1.9^b^	35.0 ± 3.4^c^	26.2 ± 1.9^c.d^
EAFTG 125 mg/kg BW	55.0 ± 3.1^a.b^	118.4 ± 4.8^b.c^	5.8 ± 1.5^a^	4.4 ± 1.3^a^	19.2 ± 1.3^a^	18.4 ± 3.0^a.b^
EAFTG 250 mg/kg BW	58.0 ± 2.9^a.b^	127.6 ± 4.8^c^	8.4 ± 1.1^a.b^	5.2 ± 1.9^a^	24 ± 2.2^a.b^	25.8 ± 2.8^c.d^
EAFTG 500 mg/kg BW	70.2 ± 3.8^c^	149.4 ± 5.6^d^	12.6 ± 2.1^b.c^	6.2 ± 2.6^a^	34.4 ± 2.3^c^	28.0 ± 5.3^d^

The different superscript letters showed significant differences between groups using One Way ANOVA, 95%, and L.S.D. posthoc.

The number of lymphocytes and neutrophils in the EETG at 500 mg/kg BW rhizome obtained the highest results compared to the other two doses. It was significantly different from the negative control. Meanwhile, the number of monocytes was the highest and significantly different (p < 0.05) with the positive and NCG. The EAFTG rhizome at a dose of 500 mg/kg BW had the highest number of lymphocytes, monocytes, and neutrophils compared to other doses and were significantly different (p < 0.05) compared to the negative control group. The increase in the number of lymphocytes, monocytes, and neutrophils in the peripheral blood of mice after carbon injection indicated an increase in phagocytic activity. This is proportional to the increase in the value of the phagocytosis constant and index on the carbon clearance method.

Generally, cells that play a vital role in the phagocytosis process are lymphocytes, monocytes, and neutrophils [36]. Another study [19] regarding the immunomodulatory activity test using the carbon clearance method, revealed an increase in the number of lymphocytes, monocytes, and neutrophils in extracts that had immunostimulant activity. The therapeutic effect of curcumin is mediated through its immune-stimulating effect, which is indicated by an increase in the number of neutrophils in the peripheral blood [37]. The increase in the number of monocytes was caused by curcumin stimulation which functions as an immunostimulant [38]. An in vivo study in a mouse model revealed that curcumin could increase the population of CD8^+^ T cells and N.K. cells. The immunomodulating effect of curcumin on CD8^+^ and CD4^+^ T cell subsets has been frequently reported in previous studies [39].

Our research is the first report about the activity of *C. heyneana* as an immunomodulator. A previous study revealed that TRPV1 is expressed in immune cells such as macrophages and dendritic cells, lymphocytes, monocytes, natural killer (NK) cells, and neutrophils [15]. The expression of TRPV1 in T-cells revealed a relationship with T cell-mediated inflammatory diseases. TRPV1 activation mediated TCR-induced calcium influx and stimulated T-cell production [16]. Some studies revealed that TRPV1 might be involved in immune cases, such as inflammatory bowel disease [40] and breast carcinoma [41]. Even though the role of TRPV1 is not clearly understood, it is promising to study this further. In order to be able to get a precise mechanism for how TRPV1 involves in our immune system. Further study in the disease related to immune abnormality is also important to ensure the activity of EETG and EAETG as immunomodulators and perform another assay, such as cytokine levels.

## 4 Conclusions

Based on these in silico results, curcumin had the best affinity to TRPV1. In vivo study using mice concluded that the ethanol extract of *Curcuma heyneana* Valeton and Zijp (EETG) has immunostimulating activity by increasing the value of the phagocytosis index and leukocyte cell count, while the ethyl acetate fraction of *C. heyneana* (EAFTG) has immunosuppressant activity at 125 mg/kg BW and immunostimulating activity with an increasing dose.

## Author contribution statement

Fifteen Aprila Fajrin: Conceived and designed the experiments; Analyzed and interpreted the data; Wrote the paper.

Melanny Ika Sulistyowaty: Conceived and designed the experiments; Contributed reagents, materials, analysis tools or data; Wrote the paper.

Mohammad Labib Ghiffary: Contributed reagents, materials, analysis tools or data.

Swara Adla Zuhra, Wulan Rosa Panggalih, Dwi Koko Pratoko: Performed the experiments.

Fransiska Maria Christianty: Analyzed and interpreted the data.

Katsuyoshi Matsunami, Anastasia Wheni Indrianingsih: Analyzed and interpreted the data; Wrote the paper.

## Funding statement

Dr Melanny Sulistyowaty was supported by 10.13039/501100014823Directorate of Research and Community Service, Ministry of Research and Technology, Indonesia [397/UN3.15/PT/2021].

## Data availability statement

Data included in article/supp. material/referenced in article.

## Declaration of competing interest

The authors declare no conflict of interest. The funders had no role in the design of the study; in the collection, analyses, or interpretation of data; in the writing of the manuscript, or in the decision to publish the results.
